# Preliminary Experience in Post-COVID-19 Mycoses: A Pathologist’s Perspective

**DOI:** 10.7759/cureus.30339

**Published:** 2022-10-15

**Authors:** Vaishali Walke, Erukkambattu Jayashankar, Ujjawal Khurana, Hemlata Panwar, T Karuna, Vikas Gupta, Neelkamal Kapoor

**Affiliations:** 1 Pathology and Laboratory Medicine, All India Institute of Medical Sciences, Bhopal, IND; 2 Microbiology, All India Institute of Medical Sciences, Bhopal, IND; 3 Otorhinolaryngology, All India Institute of Medical Sciences, Bhopal, IND

**Keywords:** covid-associated mucormycosis, aspergillosis, angioinvasion, tissue necrosis, invasive fungal infection

## Abstract

Background

Coronavirus disease is caused by the severe acute respiratory syndrome coronavirus-19. Because of co-morbidities and indiscriminate use of steroids and antibiotics, the incidence of opportunistic fungal infections has increased in COVID-affected individuals.

Aims and objectives

The aim of the study is to analyze the various tissue reaction patterns of COVID-19-associated mucormycosis in the surgical debridement specimens using routine hematoxylin and eosin (H&E) stain and special stains like periodic acid-Schiff (PAS), Grocott-Gomori's methenamine silver (GMS), Masson trichrome (MT) and Prussian blue (PB), and to understand the pathogenesis of COVID-19 sequelae.

Materials and methods

This retrospective observational study was conducted after the approval from the Institute Human Ethical Committee (IHEC) on 45 tissue samples of COVID-associated mucormycosis using routine H&E and histochemical stains such as PAS, GMS, MT, and PB. Detailed demographic profiles, clinical information, radiological findings, and relevant microbiological data in available cases, like reports on potassium hydroxide (KOH) mount preparation, and fungal culture reports on Saboraud's Dextrose Agar (SDA) medium were collected. The different histomorphological tissue reaction patterns were observed and analyzed.

Results

All the surgical debridement specimens from post-COVID cases had histomorphology of mucormycosis displaying broad, aseptate, ribbon-like fungal hyphae with right-angle branching (45/45). Six of the 45 cases also reveal thin, narrow septate, acute angle branching hyphae, indicating co-existing Aspergillosis (6/45). The histological tissue reaction patterns observed were categorized as extensive tissue necrosis (100%), vascular proliferation (82%), angioinvasion (58%), giant cell reaction (53%), fibrin thrombi (47%), septic thrombi and angiodestruction (40%), fungal osteomyelitis (33%), necrotizing granulomas (31%).

Conclusion

This study infers that post-COVID-19 associated mucormycosis, alterations in the local tissue microenvironment are found to have a favorable effect on colonizing fungi and result in destructive tissue reactions such as angioinvasion, angiodestruction, necrosis, necrotizing granulomas, suppurative inflammation, and iron pigment deposition. The spectrum of morphological changes reflects the host's immune status.

## Introduction

The COVID-19 pandemic is caused by the β-coronavirus, and the first case of severe acute respiratory syndrome coronavirus 2 (SARS-CoV-2) was reported in December 2019 in Wuhan, Hubei Province, China. In the pre-COVID era, the incidence of mucormycosis in general population ranged from 0.005 to 1.7 per million [1]. The COVID-19 pathogenesis is characterized by an altered immune response as well as an altered oxidant-antioxidant balance with increased reactive oxygen species (ROS) [2]. This pathogenic effect causes epithelial and endothelial damage, as well as multiorgan involvement later in the process. The COVID infection allows the natural commensals, Mucorales, to enter the underlying stroma through mucosal damage caused by the virus during infection and thrive best in an iron-rich microenvironment, and displays different tissue reactions because of its unique property of angioinvasion [3,4].

With this background, and the increased prevalence of COVID-19-associated mucormycosis, and to understand the pathogenesis of COVID-19, the present study was conducted to investigate the diverse histomorphological features of rhino orbital mucormycosis, and its sequelae affecting the host's immune status.

The study included examining the spectrum of mycosis in all surgical debridement specimens clinically suspected of post-COVID-19 mucormycosis, the various histomorphological tissue reactions in post-COVID-19 fungal infections of the head and neck region, and examining the role of histochemical stains such as periodic acid-Schiff (PAS), Grocott-Gomori’s methenamine silver (GMS), Prussian blue (PB), and Masson's trichrome (MT).

## Materials and methods

After receiving approval from our Institute Hospital Ethical Committee, we conducted a retrospective observational study, on 45 cases of COVID-19-associated mucormycosis (May 15th, 2021 to June 15th, 2021), who admitted to the hospital, All India Institute of Medical Sciences in Bhopal, Madhya Pradesh, India, with fungal rhino-orbital sinusitis symptoms. All surgical debridement specimens received in the histopathology lab were examined. The entire demographic profile, clinical data, and radiological information were also collected.

The surgical debridement specimens of COVID-19 cases including endoscopic removal, partial maxillectomy, and orbital exenteration were received and grossed after taking appropriate measures of COVID-19 protocol. Routine H&E and special histochemical stains like PAS, GMS, and PB were performed. Microbiological data like potassium hydroxide (KOH) mount preparation and culture information were collected in available cases.

Statistics

An Excel sheet (Microsoft, Redmond, Washington) was used to collect patients' clinical data, various histopathological findings, and special stain data for COVID-19-associated mucormycosis. Categorical data such as histomorphology features were described as percentages and analyzed.

## Results

**Figure 1:**
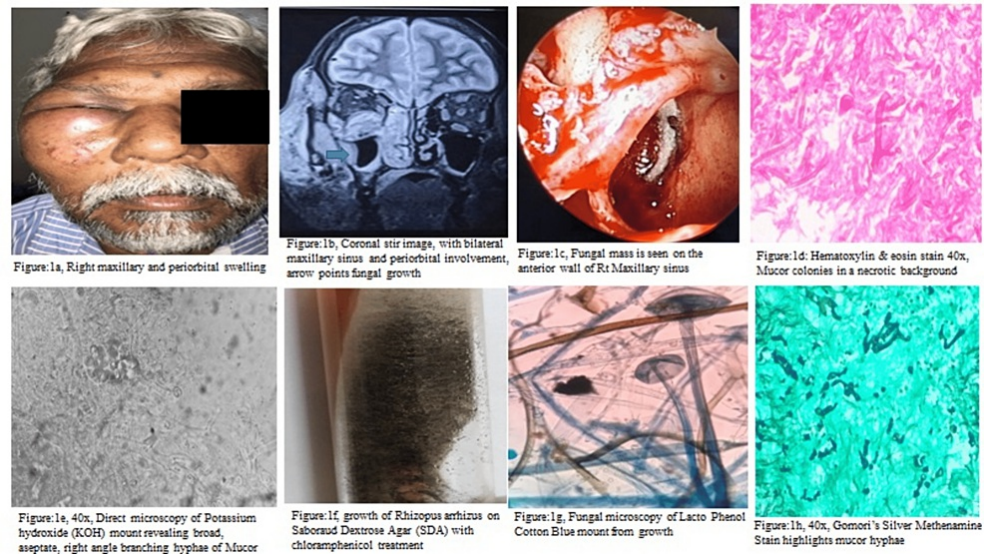
Representative images from clinical, radiological, microbiological, and histopathological features 1a: Clinical image of the right maxillary sinus and periorbital swelling; 1b: Coronal stir image with bilateral maxillary sinus and peri-orbital involvement, arrow points to fungal mass; 1c: Nasal sinus endoscopy image of anterior wall of the right maxillary sinus revealing fungal growth; 1d: Hematoxylin & eosin stain images, 40x, revealing fungal hyphae structures in the necrotic background; 1e: Direct fungal microscopy 40x, potassium hydroxide (KOH) mount preparation revealing Mucor characterized by broad, aseptate hyphae with right angle branching; 1f: Growth of *Rhizopus arrhizus* sp. on Sabourauds dextrose agar (SDA) medium treated with chloramphenicol;  1g: Fungal direct microscopy of lactophenol cotton blue mount from fungal growth; 1h: Gomoris silver methenamine stain, 40x, revealing mucormycosis hyphae

The current study included 45 cases of histopathologically confirmed COVID-19 associated mucormycosis out of 57 suspected cases of post-COVID mycoses, comprising 33 males and 12 females, with a male to female ratio of 2.8:1. The age ranged from 32 to 79 years, with the majority occurring in the fifth to sixth decades. There were 40 endoscopic removal samples and five major surgeries, which included orbital exenteration and partial maxillectomy. The post-COVID period ranged from five days to 30 days, with a mean of 8.75 days. All of these patients had a prior history of hospitalization, and the majority of them received steroid treatment in the form of dexamethasone/ methylprednisolone. Twenty-one of the 45 patients had comorbidities such as diabetes mellitus (DM) alone in 18, and both DM and hypertension in three cases. The clinical examination revealed evidence of rhino-nasal-orbital inflammation-related complaints in the form of swelling of the bilateral maxillary and periorbital regions (Figure 1a). The majority of them had inflammation involving the bilateral maxillary and ethmoid sinuses, with soft tissue involvement and, in a few cases, extension into the orbit and periorbital region, confirmed by radiological findings (Figure 1b). Sinus endoscopy revealed findings suggestive of fungal mass involving sinuses (Figure 1c). In microbiology data (Figure 1e, 1f, 1g), out of 45 cases, 36 (80%) cases had KOH mount results positive for fungal hyphae, favoring Mucor; six of these 36 cases also revealed features of aspergillosis, suggesting mixed fungal infection. Culture reports were available in 18 of 36 cases, 16 were positive for Mucor, and two were positive for double fungi. The most common species observed on culture examination were *Rhizopus oryzae* and *Rhizopus microsporus*.

**Figure 2:**
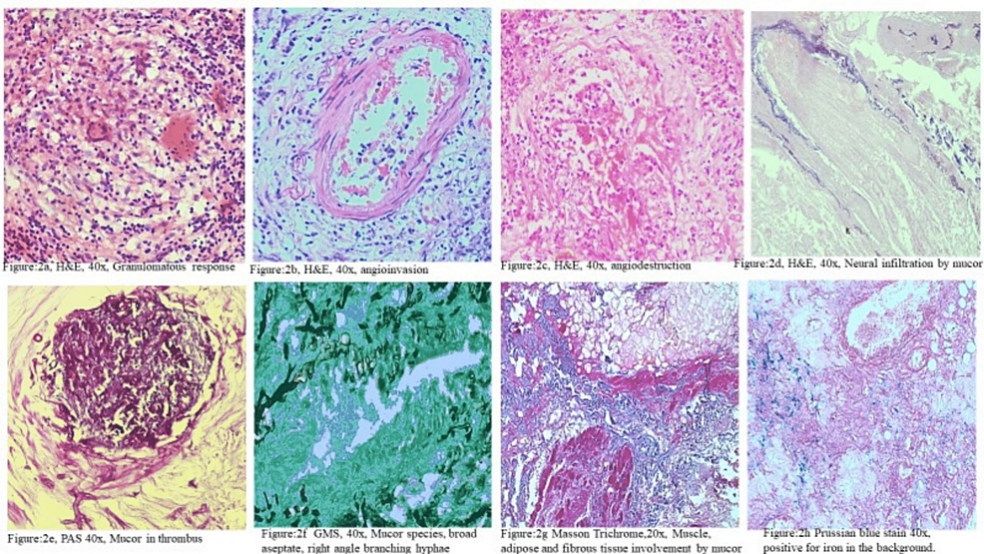
Representative images from the spectrum of tissue reaction patterns and special stains used in mucormycosis 2a: Hematoxylin and Eosin image on 40x, granulomatous response; 2b: Hematoxylin & eosin stain, 40x, revealing angioinvasion; 2c: Hematoxylin & eosin stain, 40x, revealing angiodestruction; 2d: Hematoxylin & eosin stain 40x, revealing neural bundle necrosis with evidence of perineural infiltration of fungal hyphae; 2e: Periodic acid-Schiff stain, 40x, revealing Mucor thrombi; 2f: Gomori's silver methenamine stain, 40x, mucormycosis characterized by broad, aseptate, hyphae with right-angle branching; 2g: Masson trichrome stain 40x, revealing soft tissue necrosis of muscle, and adipose tissue necrosis with  evidence of mucor hyphae infiltration; 2h: Prussian blue stain, 40x, positive of iron stores

The spectrum of histopathological findings (Figures 1d, 1h, 2) observed in the present study on routine H&E and special stains like PAS, GMS, and PB. All 45 cases of mucormycosis associated with COVID-19 had histomorphology of broad, aseptate, ribbon-like fungal hyphae with right-angle branching (45/45). Six of 45 cases also had thin, narrow septate, acute angle branching hyphae, indicating aspergillosis (6/45). The gamut of histomorphological features ranged from extensive tissue necrosis (100%), vascular proliferation (82%), angioinvasion (58%), giant cell reaction (53%), fibrin thrombi (47%), septic thrombi and angiodestruction (40%), fungal osteomyelitis (33%), necrotizing granulomas (31%), and others as mentioned in Table 1. On follow-up, it was found that five patients (5/45) died due to their illness, the postulated reason being elderly age and accompanying comorbid conditions, while the remaining patients were doing well.

**Table 1:** Histomorphological features observed in post-COVID-19 mycoses cases Different histomorphological features expressed in percentages

S.No	Histological features	Number of cases with observation	Percentage %
1	Necrosis	45	100
2	Vascular proliferation	37	82
3	Angioinvasion	26	58
4	Giant cell reaction	24	53
5	Suppurative inflammation	21	47
6	Fibrin thrombi	21	47
7	Septic thrombi	18	40
8	Angiodestruction	18	40
9	Granulomas	16	36
10	Bile pigment	16	36
11	Fungal osteomyelitis	15	33
12	Necrotizing granulomas	14	31
13	Perineurial invasion	7	15
14	Fat necrosis	4	8
15	Neural necrosis	1	1
16	Viable area	41	91

## Discussion

The coronavirus disease pandemic is caused by severe acute respiratory syndrome coronavirus 2 (SARS-CoV-2); the first case was reported in Wuhan, Hubei Province of China, in December 2019. In the general population, in the pre-COVID era, the incidence of mucormycosis is very low, varying from 0.005 to 1.7 per million. In the 2003 outbreak of SARS-CoV-2 infection, the incidence of fungal infection was 14.8-27%, and it was the main cause of death for severe acute respiratory syndrome patients, accounting for 25-73.7% of all causes of death [1].

The pathogenesis of COVID-19 is characterized by the altered immune response, and altered oxidant-antioxidant balance, with increased reactive oxygen species (ROS), cellular damage is ensured by lipid peroxidation, deoxyribose nucleic acid (DNA) damage, and protein digestion. This effect is enhanced by increased cytokine storm and hyperinflammation, associated with elevated D-dimers, interleukins (ILs), and tumor necrosis factor-alpha (TNFα) [2]. This pathogenic effect results in epithelial and endothelial damage and later multiorgan involvement. The fungi, Mucorales, are normal commensals in the upper airway. By mucosal damage during infection, the virus enters the underlying stroma and thrives best in an iron-rich microenvironment. Figure 2h highlights the presence of increased iron stores with the Prussian blue test. Also, because of its unique property of angioinvasion, the fungal infection results in thrombi formation (Figure 2e), occlusion of small vessels, and tissue ischemia followed by necrosis (Figure 2b,2c,2d) [3,4].

Prior to COVID-19, there were very few articles published in various journals about the histopathological features of mucormycosis. During the second wave of COVID-19, the most common virulent strain of COVID was the Delta variant (B.1.617.2), which was associated with a post-COVID complication "black fungus", a colloquial term for mucormycosis, an aggressive and opportunistic fungal infection [5,6].

Goel et al. [7] studied 33 cases of histopathological samples of zygomycosis over a seven-year period to better understand the rate of opportunistic infection caused by Mucorales fungi. Fungal load in the tissue (graded as mild, moderate, or severe), fungus mean diameter, neutrophilic and granulomatous response, tissue invasion, and necrosis were all graded and evaluated for prognostic significance. Their study quoted that angioinvasion is the most critical histopathological finding associated with poor prognosis [7]. In the present study, tissue necrosis, followed by angioinvasion, was associated with morbid features (Table 1).

In the current study, all the patients (45/45) had tissue evidence of mucormycosis; in addition, aspergillosis was identified in six cases suggesting mixed fungal infection.

Mucor is distinguished histologically by the presence of broad irregular, pauci-septate, ribbon-like fungal hyphae with right-angle branching (Figure 1d, 1e, 1h). Aspergillosis is distinguished by thin septate, fungal hyphae with an acute angle branching. Similarly, mixed fungal infection is also reported by Kandswamy et al. in 8/58 cases, but they have observed a combination of *Aspergillus* sp. and *Candida* sp. [8].

According to the available post-COVID literature, [9-16] India is the epicenter of black fungus, primarily mucormycosis, as compared to more prevalence of aspergillosis in the Western world. Reasons include rampant usage of steroids and associated risk factors like diabetes mellitus and environmental conditions. COVID-associated invasive fungal sinusitis (CAIFS) is a term used to describe fungal sinusitis caused by COVID-19 [10].

Similar to the current study, Jain et al. [17] observed different histological findings in post-COVID-19 mycoses and graded amount of fungal load, tissue necrosis, and angioinvasion, which were correlated with poor prognosis. All cases in the present series had tissue necrosis which corresponds to the unique angioinvasive property of Mucor. Five patients had presented with marked tissue necrosis evidence and had undergone extensive tissue debridement, including maxillectomy and orbital exenteration.

Frater et al. [18] examined the histopathology of invasive zygomycosis in 20 cases, of which 55% were of rhino-cerebral type. The histopathological patterns observed include inflammatory responses predominantly neutrophilic in 50%, granulomatous in 5%, and pyogranulomatous in 25%. The invasive disease was characterized by prominent infarcts (94%), angioinvasion (100%), and, surprisingly, prominent perineural invasion (90%) in biopsies that contained nerves for evaluation. In the present study, all the cases were of rhino orbital mucormycosis in post-COVID setup. The gamut of histopathological features in the current study ranged from suppurative inflammation in 47% of cases, granulomatous inflammation in 36% of cases, and suppurative granulomatous inflammation in 31% of cases. The dominant histological finding was necrosis, similar to other studies, followed by angioinvasion and destruction. Sundaram et al. [19] studied 30 cases of non-COVID-19 rhino-cerebro-orbital mucormycosis cases and identified perineural invasion as more commonly associated with angioinvasion and necrosis, wherein the present study exhibited perineural invasion in only 15% of cases. From the available literature, it's understood that the main neurological manifestation is in the form of hyposmia, anosmia, and visual disturbances due to the neurotoxic effect of the virus. Poutoglidis et al. [20] reported bilateral vocal fold palsy as a rare neurological manifestation in a post-COVID-19 case who was intubated for a prolonged interval and stayed in the intensive care unit. With prolonged intubation and in the presence of COVID-associated inflammation, the patient had developed cricoarytenoid joint fixation resulting in bilateral vocal fold palsy. So, patients with prolonged ICU stay with intubation must be evaluated with laryngeal electromyography.

Challa et al. [21], in their study on non-COVID mycoses, concluded that histopathology plays a major role in the diagnosis of infection due to filamentous fungi, especially when cultures were not submitted or negative. The discrepancy between histological and culture diagnosis was either due to dematiaceous fungi being interpreted as *Aspergillus* species or probable dual infection. Similarly, the present study's histopathological diagnosis was well supported by KOH positive reports for fungal hyphae in 36, and by culture in 18 cases, of which 16 cases showed growth of *Rhizopus oryzae* and *Rhizopus microsporus*, two cases had grown mixed fungal hyphae. Microbiology correlation was not available in nine cases.

In the present study, the male-to-female ratio and the age group involved were also similar to other published literature [7,17-19,21].

Although the standard H&E stain can detect fungal disease, which appears as pale hyphal structures, we used special stains like PAS, GMS, MT, and Prussian blue to highlight the presence of fungal infection in the background of necrosis. MT can show in detail the soft tissue involvement such as adipose tissue necrosis, skeletal muscle necrosis, angioinvasion, and angiodestruction, and the presence of an iron-rich tissue microenvironment can be highlighted by PB stain, which is more conducive to fungal growth. We can infer that using ancillary techniques like GMS, PAS, and MT improves the identification of different tissue reaction patterns in invasive fungal infections [22].

In the follow-up of surgically treated cases, the mortality rate of five out of 45 cases was noted, the reason being elderly age associated with other comorbid conditions, like diabetes, and excessive tissue involvement by the fungal infection, characterized by necrosis, angioinvasion, angiodestruction, and necrotizing granulomas.

Limitations of the study

Because the study approval is only valid for a limited time, the sample size is limited to 45 cases. The molecular workup for the fungi, specific categorization of mucor species, and molecular data related to the COVID-19 strain were not included in the study due to financial constraints.

## Conclusions

In the Indian setting, COVID-associated rhino-orbital mucormycosis was the most common invasive opportunistic fungal infection, outnumbering invasive fungal aspergillosis or candidiasis. It is distinguished by widespread tissue necrosis, angioinvasion, suppurative granulomas, vascular proliferation, fibrin, and septic thrombi. The study also emphasized the role of pathologists in identifying the destructive tissue reaction caused by fungal species, thereby assisting clinicians in effective patient management and prognosis.

## References

[1] Singh Y, Ganesh V, Kumar S, Patel N, Aggarwala R, Soni KD, Trikha A (2021). Coronavirus disease-associated mucormycosis from a tertiary care hospital in India: a case series. Cureus.

[2] Cevik M, Kuppalli K, Kindrachuk J, Peiris M (2020). Virology, transmission, and pathogenesis of SARS-CoV-2. BMJ.

[3] Pincemail J, Cavalier E, Charlier C (2021). Oxidative stress status in COVID-19 patients hospitalized in intensive care unit for severe pneumonia. A pilot study. Antioxidants (Basel).

[4] Ntyonga-Pono MP (2020). COVID-19 infection and oxidative stress: an under-explored approach for prevention and treatment?. Pan Afr Med J.

[5] Balushi AA, Ajmi AA, Sinani QA (2022). COVID-19-associated mucormycosis: an opportunistic fungal infection. A case series and review. Int J Infect Dis.

[6] Rao VU, Arakeri G, Madikeri G, Shah A, Oeppen RS, Brennan PA (2021). COVID-19 associated mucormycosis (CAM) in India: a formidable challenge. Br J Oral Maxillofac Surg.

[7] Goel A, Kini U, Shetty S (2010). Role of histopathology as an aid to prognosis in rhino-orbito-cerebral zygomycosis. Indian J Pathol Microbiol.

[8] Kandasamy S, Muthuraju S, Vasugi A, Chandrasekar M, Murugan R, Inbasekaran P, R P (2022). Clinicopathological study of mucormycosis in COVID-19 patients: experience from a tertiary care center in South India. Cureus.

[9] Muthu V, Rudramurthy SM, Chakrabarti A, Agarwal R (2021). Epidemiology and pathophysiology of COVID-19-associated mucormycosis: India versus the rest of the world. Mycopathologia.

[10] Song G, Liang G, Liu W (2020). Fungal co-infections associated with global COVID-19 pandemic: a clinical and diagnostic perspective from China. Mycopathologia.

[11] Baddley JW, Thompson GR 3rd, Chen SC (2021). Coronavirus disease 2019-associated invasive fungal infection. Open Forum Infect Dis.

[12] Kant R, Totaganti M, Mohan B (2022). Clinical characteristics of 100 patients with COVID-19-associated mucormycosis from a tertiary care center in North India. Cureus.

[13] Sharma S, Grover M, Bhargava S, Samdani S, Kataria T (2021). Post coronavirus disease mucormycosis: a deadly addition to the pandemic spectrum. J Laryngol Otol.

[14] Kamath S, Kumar M, Sarkar N, Ahmed T, Sunder A (2022). Study of profile of mucormycosis during the second wave of COVID-19 in a tertiary care hospital. Cureus.

[15] Mehta S, Pandey A (2020). Rhino-orbital mucormycosis associated with COVID-19. Cureus.

[16] Vijapur MM, Kattimani V, Varsha VK, Girish HC, Kamat M, Ram B (2022). COVID-19 associated mucormycosis (CAM): a single hospital-based study. J Oral Maxillofac Pathol.

[17] Jain K, Surana A, Choudhary TS, Vaidya S, Nandedkar S, Purohit M (2022). Clinical and histology features as predictor of severity of mucormycosis in post-COVID-19 patients: an experience from a rural tertiary setting in Central India. SAGE Open Med.

[18] Frater JL, Hall GS, Procop GW (2001). Histologic features of zygomycosis: emphasis on perineural invasion and fungal morphology. Arch Pathol Lab Med.

[19] Sravani T, Uppin SG, Uppin MS, Sundaram C (2014). Rhinocerebral mucormycosis: pathology revisited with emphasis on perineural spread. Neurol India.

[20] Poutoglidis A, Tsetsos N, Karamitsou P, Forozidou E, Garefis K, Keramari S, Vlachtsis K (2022). Bilateral vocal fold palsy following COVID-19 infection. Ear Nose Throat J.

[21] Challa S, Pamidi U, Uppin SG, Uppin MS, Vemu L (2014). Diagnostic accuracy of morphologic identification of filamentous fungi in paraffin embedded tissue sections: correlation of histological and culture diagnosis. Indian J Pathol Microbiol.

[22] Challa S, Sistla R (2022). Histopathology diagnosis of filamentous fungi. Curr Fungal Infect Rep.

